# Raman Spectroscopy for Instant Bladder Tumor Diagnosis: System Development and In Vivo Proof-Of-Principle Study in Accordance with the European Medical Device Regulation (MDR2017/745)

**DOI:** 10.3390/cancers16183238

**Published:** 2024-09-23

**Authors:** Ines Latka, Karin Mogensen, Florian Knorr, Cansu Kuzucu, Florian Windirsch, Dragan Sandic, Jürgen Popp, Gregers G. Hermann, Iwan W. Schie

**Affiliations:** 1 Leibniz-Institute of Photonic Technology (IPHT), Leibniz-Health-Technologies, Leibniz-Center for Photonics in Infection Research (LPI), Albert-Einstein-Str. 9, 07745 Jena, Germany; 2 Urology Department Herlev, Gentofte Hospital, Borgmester Ib Juuls vej 23A, DK-2730 Herlev/Copenhagen, Denmark; 3 2M Engineering, John F Kennedylaan 3, 5555XC Valkenswaard, The Netherlands; 4 Blazejewski MEDI-TECH GmbH, Rheinstr. 1, 793650 Freiburg, Germany; 5 Institute of Physical Chemistry (IPC) and Abbe Center of Photonics (ACP), Leibniz Center for Photonics in Infection Research (LPI), Friedrich-Schiller-University Jena, Helmholtzweg 4, 07743 Jena, Germany; 6 Department of Medical Engineering and Biotechnology, University of Applied Sciences Jena, Carl-Zeiss-Promenade 2, 07745 Jena, Germany

**Keywords:** Raman spectroscopy, bladder cancer, clinical study, medical device regulation (MDR)

## Abstract

**Simple Summary:**

Raman spectroscopy is a label-free optical method that has proven its suitability for the discrimination between cancerous and noncancerous tissue. Bladder cancer diagnosis typically relies on excisional biopsies and histopathology, which are considered the gold standard. Here, we want to use an endoscopic setup to demonstrate the applicability of Raman spectroscopy in a clinical in vivo setting. In the European Union, the regulatory framework is given by the Medical Device Regulation. We describe the path and the necessary effort to be able to conduct a clinical investigation within this framework. The aim of this clinical examination was to perform Raman spectroscopy in the living bladder and to record Raman spectra of normal and tumorous bladder tissue and to determine the degree of bladder tumor in a post-processing step. The open prospective study includes 21 patients and presents preliminary results for the diagnostic abilities of our approach.

**Abstract:**

This work reports on an in vivo Raman-based endoscopy system, invaScope, enabling Raman measurements of healthy and tumor bladder tissue during an endoscopic procedure in the operating theatre. The presented study outlines the progression from the initial concept (validated through previously performed ex vivo studies) to the approval and implementation of a clinical investigational device according to the requirement within the framework of the European Medical Device Regulation (MDR2017/745). The study’s primary objective was to employ the invaScope Raman system within the bladder, capturing in vivo spectroscopic Raman data followed by standard histo- and cytopathological examinations of urological tissue (considered the gold standard). The collected data were analyzed and correlated with histopathological findings post-procedure. Additionally, the study aimed to assess the feasibility of using diagnostic equipment, probes, and software for application in a clinical setting, evaluating usability aspects that are important during surgical procedures. This research represents a pivotal step toward advancing Raman spectroscopy for routine clinical use in characterizing bladder lesions.

## 1. Introduction

The diagnosis of bladder cancer typically relies on excisional biopsies and histopathology, which are considered the gold standard. When white light cystoscopy and positive urinary cytology strongly indicate bladder cancer, a transurethral resection of a bladder tumor (TURBT) is often necessary, combining both diagnostic and therapeutic functions. During the TURBT, the surgeon removes the tumor for histological evaluation to confirm the diagnosis, determine the cancer stage and grade, and provide treatment by removing visible tumors [1,2,3]. One improvement in this is a photodynamic-guided bladder biopsy using blue light cystoscopy. Hexaminolevulinate (Hexvix) [4] is a photosensitizer that is administered prior to examination and accumulates only in cancer cells. Illumination with UV light causes the photosensitizer to fluoresce, making it visible to the physician. It is recommended for the detection of non-muscle invasive bladder cancer (NMIBC), especially carcinoma in situ (CIS) [3], a flat, high-grade, non-invasive urothelial cancer. It can outperform white light cystoscopy, as white light has a relatively high false positive rate [4,5].

However, due to histological preparations, the delay in diagnosis (up to 7 days) affects timely treatment initiation, impacting patient outcomes and healthcare costs. As the diagnosis is not available instantly, effective treatment may be delayed. Early and immediate diagnosis would enable the earlier initiation of effective treatment, thereby reducing cancer progression, recurrences, and the need for follow-up procedures. This, in turn, may increase survival rates and have a significant impact on public health by reducing costs and improving quality of life [6]. This applies not only to bladder cancer but to most tumors. Thus, there is a great need for new technologies that can precisely localize and characterize a tumor to ensure complete removal. A direct and reliable classification enables the initiation of an individual therapy plan tailored and applied to the patient as quickly as possible. In recent years, Raman spectroscopy has developed from a purely scientific research method in chemical analysis into a potential tool for clinical research [7,8,9,10,11,12] that enables label-free intraoperative diagnostics by providing instant tumor identification or even grading without invasive procedures. Previous ex vivo studies on bladder tumors suggest Raman spectroscopy’s ability to differentiate between tumor and non-tumor tissue and determine tumor aggressiveness [12,13,14,15,16,17,18,19,20,21]. There are recent publications using fiber optic probes during in vivo clinical studies [22,23]. Both use commercial fiber probes from EMVision LLC and do not include the high wavenumber region, even though this region provides very relevant bands for classification. In [22], a commercial system is used and combined with cryoablation. However, it does not include any mention of the sterilization procedure or any regulatory hurdles for the study. Moreover, the presented spectra have a strong resemblance to the illumination spectrum of the endoscopy unit. In [23], superficial and non-superficial probes are used to compare their discriminating power.

Nevertheless, despite promising ex vivo results, the clinical translation to an in vivo study bears a different set of requirements [24,25,26,27,28,29,30,31] which must be fulfilled. Those requirements include designated fiber optical probes and the appropriate hard- and software, which are suitable for clinical investigational studies. Most importantly, in the European Union, the conduction of a clinical investigational study is regulated by the new European Medical Device Regulation (MDR [32]), which defines the requirements for all medical devices intended to be used for human beings, with the purpose of inter alia diagnosis, prevention, monitoring, prediction, prognosis, treatment, or alleviation of disease. Overall, the EU’s MDR represents a tightening of the regulations for medical devices, with the overarching aim to increase safety with a clear focus on the patients. The MDR also applies to clinical investigations that are conducted for studies’ research purposes, e.g., for translational research. Article 82 of the MDR sets the rules for “other clinical investigations”. As such, the regulation specifically applies to the fiber optical probe and the hard- and software, which must be used in a clinical study.

Here, we report on the design and development of an endoscopic Raman probe, the backend unit, and the software for an in vivo clinical investigation in compliance with the MDR. To achieve such a compliance, we have implemented a mechanically robust single-arm fiber probe that can be plugged into the backend device without further alignment. The probe can also be plasma (hydrogen peroxide)-sterilized according to standard clinical procedures. Moreover, we have developed the corresponding hardware and software according to the applicable standards to satisfy the outlined requirements set out in the MDR. We describe our transition from multiple successful ex vivo studies to a clinical investigation by translating the invaScope to real-time in vivo measurements during TURBT procedures. The study aims to validate the system performance and usability in an in vivo setting and to correlate spectroscopic data with histopathological results, enabling urologists to effectively characterize bladder lesions. This approach could enable earlier, more accurate diagnosis, potentially improving patient outcomes and reducing healthcare costs.

The manuscript is structured as follows: first, we describe the regulatory hurdles and the resulting conditions that must be considered when designing the invaScope system. This is followed by a detailed description of the probe’s technical aspects, including the recording software, and the clinical study for the in vivo examination of bladder tumors.

## 2. Materials and Methods

### 2.1. Regulatory Aspects

**Figure 1:**
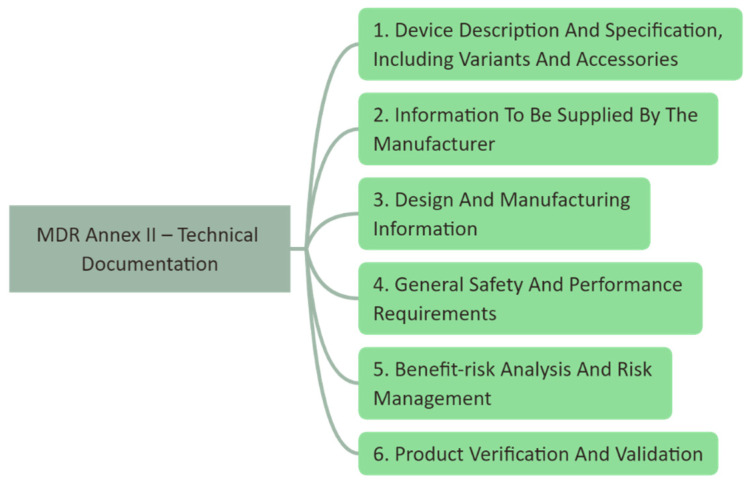
Structure of MDR Annex II, which determines the required content of the technical documentation. In accordance with the MDR requirements, these aspects must be considered, implemented, and documented to perform a clinical investigative study.

**Figure 2:**
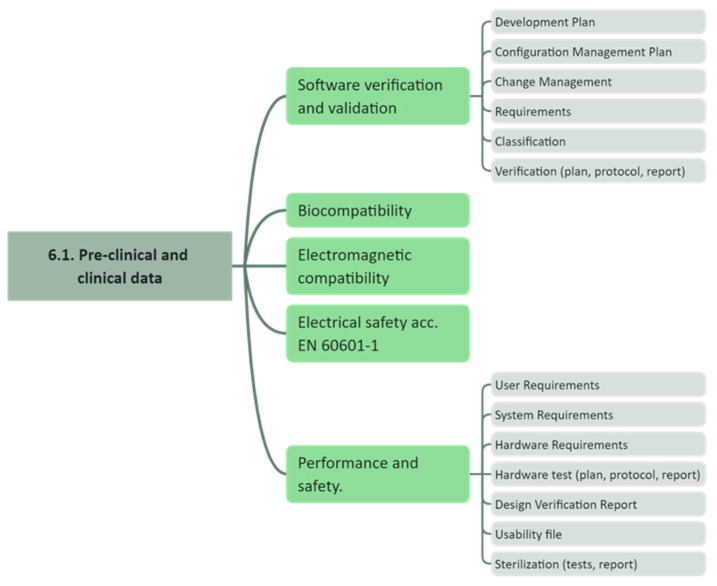
Exemplary detailed view on a sub-item of the requirements for the technical documentation, which will result in individual documents that have to thoroughly describe the implementation of the individual items.

The clinical study conducted here is subject to Article 82 of the MDR, summarized under the term “other clinical investigations”, which are primarily intended to answer scientific or other questions and which are not part of a systematic and planned process of product development or product observation by a current or future manufacturer. The most important criterion here is that the device fulfills the general safety and performance requirements (GSPR, Annex I of the MDR), except for the aspects covered by the clinical investigation (Article 62(4) bullet I of the MDR). The technical documentation according to Annex II of the MDR is used to give proof for the fulfillment of the GSPR. The necessary effort is strongly dependent on the intended purpose of the device, as this influences the classification of the device. Annex XIII of the MDR contains a set of rules for the classification. The higher the class, the higher the risk and the greater the effort required. Figure 1 shows the structure of Annex II to give an indication of what needs to be addressed for the technical documentation. Figure 2 shows a zoom into sub-item 6.1. Pre-clinical and clinical data. This extract gives an indication of what needs to be conducted. As only a few groups have direct access to laboratories, e.g., for biocompatibility or electromagnetic compatibility, external validated laboratories must be involved.

For the specific case of the Raman invaScope reported here, requirements for risk management, user friendliness, software development, electrical safety, laser safety, and electromagnetic compatibility must be determined. The definition of the intended use is of crucial importance. Our study aims to develop a Raman system to be used in the bladder to collect spectroscopic Raman data during surgery. Based on this intended use, a detailed requirements and risk analysis was carried out to derive system, hardware, and software requirements (function, safety, usability). All these analyses are carried out to arrive at a design that ensures maximum patient safety. This includes compliance with lasers, electrical safety, biocompatibility, and reprocessing standards. In total, the requirements determined for our Raman invaScope system amount to around 50 documents and more than 600 pages.

### 2.2. Description of Raman invaScope

#### 2.2.1. Raman Fiber Probe

The part of a medical device that comes into contact with the patient is called the “applied part”. In the case of the Raman invaScope, the applied part is the Raman fiber probe. As an outcome of the regulatory requirements, the following design criteria for the Raman fiber probe have been derived:

- For easy and reliable use, the Raman fiber probe is designed as a contact probe.
- To improve usability and reduce the complexity of probe manufacturing, an unbranched design is used.
- The Raman fiber probe is designed for plug-and-play use, using a keyed snap-in linear connector with guide pins to ensure a precise alignment.
- The Raman probe parts that will come in direct contact with the patient have to be biocompatible.
- The fiber probe needs to be long enough, so that the clinician cannot touch the patient and the device at the same time.
- Laser engraved serial numbers for traceability of use are required.

**Figure 3:**
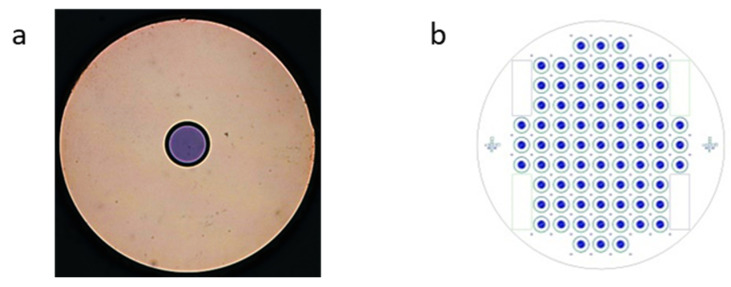
(**a**) Single filter set, consisting of an inner circle (SP) and an outer ring (LP); (**b**) wafer design with markings for precise filter singulation.

The endoscopic Raman probe is designed as an applied part of type BF. It is designed based on a 10 + 1 configuration, i.e., 10 collection fibers surrounding a single excitation fiber, together with a filter configuration to interfere with the intense Raman signal from the fiber itself [33]. Here, we use multimode optical fibers (AFS105/125 Y Low OH, Thorlabs) for the transmission of the Raman excitation laser and the collection of the generated Raman signal. To improve the collection efficiency, 10 collection fibers are used, arranged in a circle around the excitation fiber. As mentioned above, the probe is designed as a contact probe. To remove the fiber-based Raman background [12,33], a short-pass (SP) filter was used to remove the in-fiber generated background at the distal end of the excitation fiber. To prevent reflected laser light from entering the collection fibers, a long-pass (LP) filter was placed at the distal end of the collection fibers. In order to decrease the complexity for the probe assembly (e.g., inherent adjustment accuracy for both filter types), a structured filter was designed and produced (Optics Balzers Jena GmbH/Germany), as shown in Figure 3a. This means that both filter types are realized on a common quartz glass substrate (thickness 700 µm). These absorption-free multi-layer interference coatings are specifically designed for our optical arrangement (laser excitation wavelength, collection range, required optical density, edge steepness) and are very economical. One substrate (ø 50 mm) can hold up to about 70 filter sets and up to 18 substrates can be coated in one production run, as shown in Figure 3b.

The filter kit was glued to the pre-assembled probe head using an optical adhesive (UV adhesive Norland optical Adhesive 88). The probe head with the attached compound filter was protected by a stainless-steel cap. The adhesive used is medical grade (Vitralit^®^ 4731, Panacol-Elosol GmbH, Steinbach, Germany). A biocompatible silicone tubing (inner diameter 1.5 mm, outer diameter 3.4 mm, SZ111S, Witzenmann-Speck GmbH, Kieselbronn, Germany) protects the fibers and the patient over the entire length of the fiber probe (3 m). To effectively couple the collected Raman signal to the Raman spectrometer, a 12-fibre multi-fiber connector (MTP ^®^ male, US Conec, Hickory, NC, USA) with linearly arranged fibers was used. This MTP connector contained both the excitation and collection fibers. The keyed snap-in linear fiber connectors (MTP, male) with guide pins ensured precise alignment and correct orientation. This straight (unbranched) design further reduced complexity and improved the repeatability of the assembly process.

To reduce the risk of infection during the in vivo measurements in the bladder, it is paramount that the fiber optic probe can be sterilized. For practical and economic reasons, the aim was to use the probe multiple times, which is why it was defined that the probes should be sterilized at least 5 times without loss of function, i.e., laser transmission > 50%, and that the fiber optical probe remains mechanically intact. The fiber probes were sent three times for clinical sterilization tests. Each time, the sterilization process was performed 5 times on non-consecutive days. The sterilization was performed by the sterilization department at Herlev and Gentofte Hospital (urology department) using an automated process (Sterrad system). During the process, the probes were packed in a 12.5 × 12.5 × 3.5 cm box suitable for hydrogen peroxide sterilization. In the end, one probe was sterilized 5 times, one 10 times and one 15 times. Even after 15 sterilization cycles, the probe met the pass criteria, with laser transmission still at 85% of the value before sterilization. To trace the number of sterilization cycles of each probe, we engraved a unique mark into the silicone tubing using a pulsed Yb fiber laser (Leibniz-IPHT, Jena, Germany). To track any changes in characteristics, the fiber probes were thoroughly characterized before and after each set of cycles. This included the optical inspection of relevant parts, e.g., the end faces, laser transmission measurements, and Raman measurements using standard samples (polystyrene [34], SRM2241 [35]).

#### 2.2.2. Raman System

**Figure 4:**
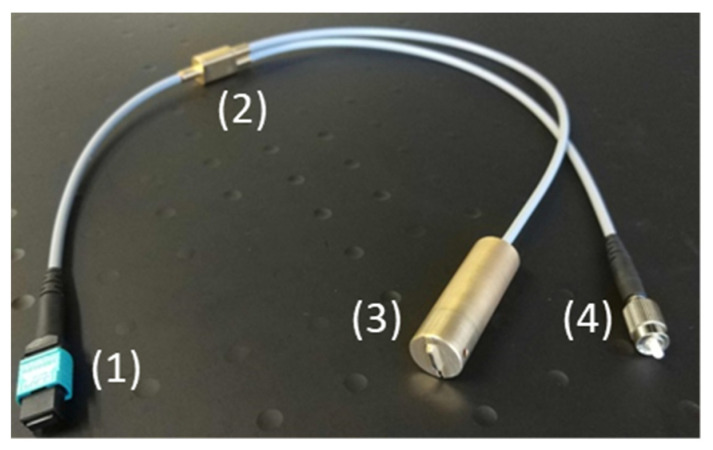
The fiber optic adapter enables an alignment-free connection between the fiber optical probe and the spectrometer and consists of (1) an MTP connector, (2) bifurcation, (3) a line connector as the spectrometer entrance slit, and (4) an FC connector for laser coupling. The fiber optic probe is connected to the MTP connector, which is integrated into the invaScope base-unit.

**Figure 5:**
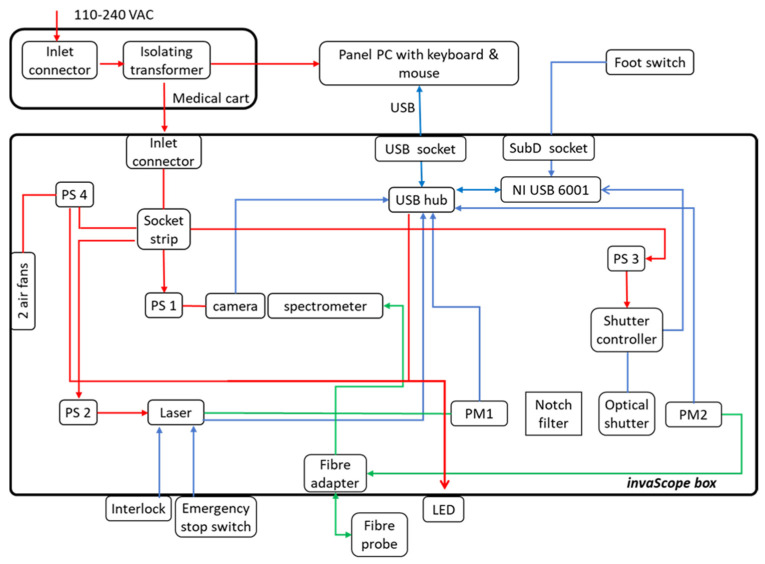
Block diagram of the invaScope is required as a part of the technical documentation and describes the connections between components integrated into the unit. Red line: power cables; blue line: data cables; green line: optical fiber connection; PS: power supply; PM: power monitor.

The Raman system used with the fiber probe contained all the necessary Raman components in a common housing. These included the Raman excitation laser (FER-785, Princeton Instruments, Trenton, NJ, USA), a CCD camera (Pixis 100, Princeton Instruments) and an imaging spectrograph (Acton Series LS 785, Princeton Instruments). To ensure an alignment-free coupling between the probe and the spectrometer, a custom-made fiber optic adaptor was developed that realizes the separation of the excitation and collection path, as shown in Figure 4. This means that the division of the optical paths is shifted to the periphery and therefore only needs to be built once. The female MTP multi-fiber connector ((1) in Figure 4) was adapted to the multi-fiber connector of the probe. The die-cast EMI (electromagnetic interference)-shielding MPO adapter (Molex, Lisle, IL, USA) in the front of the enclosure maximizes coupling strength (adapter to fiber probe). The ten collecting fibers were arranged in parallel in a silicon structure with V-shaped grooves (manufactured at Leibniz-IPHT). The fiber end faces thus formed a line ((3) in Figure 4) that can be used as the entrance slit of the imaging spectrograph (LS785 Teledyne Princeton Instruments). It is configured to fit into the mount of the spectrograph. The leg of the adapter ((4) in Figure 4) that pointed toward the excitation laser (Fer785-MM diode laser, Teledyne Princeton Instruments) was connectorized (FC/PC). An optical shutter (SHB1T, Thorlabs, Newton, NJ, USA) was used to control laser emission. Before and after the optical shutter, a small part of the laser light was redirected to an optical power meter. These power meters (PM16-120, Thorlabs) were used for laser power calibration and status control of the laser and shutter. A medical foot pedal (SK12, Steute GmbH, Löhne, Germany), which was used to execute the acquisition, was integrated into the unit. The housing of the Raman device was made of powder-coated aluminum, had a built-in EMI-shielding gasket, and had 2 fans for temperature control. It was mounted on a medical cart for easy access to the operating theatre (Compact-cart Basis “Profi” with isolating transformer, iTD GmbH, Pfarrkirchen, Germany). The isolating transformer with an earth leakage guard (ZV.9359.999, Noratel/Grafenau, Germany) provided the two means of protection against electric shock required by the standard DIN EN 60601-1:2013. The cart was equipped with a height variable support arm on its swivel arm for the used panel PC. The panel PC (Medical Line THA.leia^3^ 21.5” Touch, MCD Medical Computers Deutschland GmbH, Mönchengladbach, Germany) is compliant with EN 60601-1 and can be used in operating theatres. The mouse (Mighty mouse, Man & Machine, Landover, MA, USA) and keyboard (Really Cool Touch Keyboard, Man & Machine) were sealed, waterproof, and immersible (IP68). The block diagram of the system is depicted in Figure 5.

#### 2.2.3. Software

To control the data acquisition with the invaScope, we have developed software, the invaSoft, which includes hardware testing, initialization, guided calibration procedures, and data acquisition. Because the clinical setting is very demanding, we focused during the development on an intuitive and guided interface to ensure error-free operation. The software was developed using LabVIEW 2020. invaSoft has two selectable languages, i.e., German and English, catering to a diverse user base. At startup, the software performs a series of critical hardware checks to ensure operational integrity. These checks include detector recognition that not only detects the camera but also manages its cooling process effectively, bringing the temperature down to −75 °C. This temperature regulation is visually represented by a progress bar, enhancing user interaction. Additionally, the software recognizes and configures the laser settings by adjusting the current presets to optimize performance. It also includes the monitoring of the photodiodes that continuously monitor the laser power before and after the activation of the optical shutter, ensuring consistent output and safety during procedures. During the endoscopic procedures, invaSoft does not allow the user to modify any set parameters. This ensures that potential misuse and endangerment of the patient and medical staff are reduced. Users can monitor the status of the laser power and shutter, ensuring they are always aware of the current operating conditions. The software provides real-time device status updates and controls the shutter mechanism based on procedural requirements. To improve usability, invaSoft always loads the default settings, such as laser power and integration time, tailored to specific procedural needs. It facilitates comprehensive data collection and monitors the status of foot switches, a critical component for hands-free operation. The software ensures that calibration is performed every time a new (sterilized) fiber probe is used. The software records and displays the acquired Raman spectra and it associates these spectra with anonymized patient information and specific fiber probes. The software ensures that all Raman spectra are methodically stored in designated patient-specific folders on the hard drive, allowing for efficient data management and retrieval. During operations, the software provides both acoustic and optical feedback when the laser is emitted, aiding in procedural accuracy and safety. Administrators can adjust account settings and manage access rights, ensuring that the system remains secure and is operated only by authorized personnel.

#### 2.2.4. Risk Analysis

**Figure 6:**
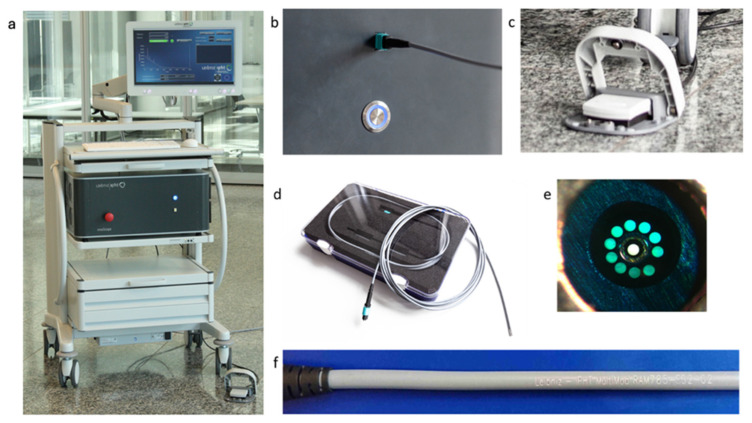
Raman invaScope for fiber optic probe-based diagnostics of cancer. (**a**) The invaScope system, presented in full and built on a medical cart for transport into the use environment. (**b**) The MTP-based plug-and-play connection between the fiber optical probe and the base unit. (**c**) A medical grade foot pedal is used to start the individual measurements. (**d**) The fiber optical probe developed at the Leibniz-IPHT. (**e**) Front view of the fiber optical probe, where the ten collection fibers circularly surround the excitation fiber. (**f**) The engraved information on the fiber optical probe to ensure the traceability.

The invaScope Raman system (Figure 6) is classified as a class IIa medical device according to Rule 10 of the MDR17/745, Annex XIII, because it is an invasive and active device with a transient use duration. It is designed as an endoscopic device capable of capturing molecular signatures of tissues during cystoscopy or a TURBT. To ensure the device’s safety, it requires a risk analysis and substantial testing. This includes ex vivo testing [17,18,19,20], electromagnetic compatibility [36,37], electrical safety [38], laser safety [39,40], biocompatibility, sterilization [41], usability [42,43], and probe processing (cleaning, disinfection, and hard- and software verification) [44]. In order to prove that our Raman system does not interfere with the operation of other devices or equipment (e.g., cardiac monitors, anesthesia machines, or cardiac pacemakers), emission tests (radiated and conducted emissions) were performed at the Accredited Laboratory for Calibration and EMC—MeßTechnik Nord GmbH (Jena, Germany). The test basis was ISO 60601-1-2 [36], which refers to CISPR 11 [37]. As a result, our investigational device was found to comply with Group 1 (e.g., medical devices) and Class A (other than domestic) limits. EN 60601-1 clause 8 Protection against electrical hazards from ME equipment and clause 16 ME systems of EN 60601-1 [38] were carried out by an independent medical engineering company specializing in the testing of medical devices (Ingenieurbüro für Medizintechnik Dresden GmbH, Dresden, Germany). The conformation for compliance with EN 60601-2-22 was performed by the same company [39,40]. Hardware and software verification were performed at Leibniz-IPHT Jena by testers who were independent of the development team. The tests verified that all hardware and software requirements were implemented and performed as intended. Once all risk-minimizing measures have been implemented and the tests described have been successfully completed, our system is ready for clinical trials.

The invaSoft software has been developed in accordance with EN 62304 as Software Safety Class A (a hazardous situation cannot arise from a failure of the software). This means that in the event of a software failure, no injury or damage to the patient’s health is possible as follows:

- In the event of software failure, no measurements can be taken with the device. There is no harm to the user or the patient.
- The device is not intended to be used for diagnosis or monitoring, i.e., the clinician is not using any information to base a medical decision based on the information from the device.
- The device does not store critical patient care data or sensitive data.
- The invaScope has no emergency, critical, or life-sustaining functions; as such, the wellbeing of the patient does not depend on the device.
- There are no software-dependent risk control measures.

## 3. Results

### 3.1. Clinical Phase I Study

We have conducted an open prospective study (ClinicalTrials.gov registration NCT05124106 (https://clinicaltrials.gov accessed on 22 September 2024)) involving participants undergoing TURBT (transurethral bladder tumor resection) or cystoscopy with a biopsy for suspected urothelial bladder tumors. The primary outcome of this investigation was defined to perform Raman spectroscopy measurements within the bladder to obtain spectra from normal and tumor tissue, and subsequently to spectroscopically differentiate between a tumor and healthy bladder wall and to determine the grade of bladder tumors in comparison to standard histo- and cytopathological examinations. The differentiation was planned to be performed post-acquisition and validated against histopathological findings. As such, the results will be spectroscopically identifiable signatures, which can be related to a disease stage using a multivariate statistical model. The secondary outcome was to evaluate the usability of the invaScope Raman probe and software when the urologist examined the bladder for lesions.

To achieve these goals, the above-described Raman invaScope system was used during the procedures at the urology department of Herlev Hospital in Copenhagen. The procedure utilized rigid endoscopes with working channels (Blazejewski-MEDI-TECH, Sexau/Germany) through which the probe can be pushed through to the object under consideration. The procedure was performed under general anesthesia, adding approximately 10–15 min to the operation time, with each measurement lasting 1–5 s.

In this study, a total of 21 participants who met the specific inclusion criteria were, after giving their informed consent, included in the clinical study. Eligible participants must have suspected bladder tumors and be scheduled for either transurethral bladder tumor resection (TURBT) or cystoscopy with a biopsy under general anesthesia. Exclusion criteria included the presence of macroscopic hematuria, pregnancy or breastfeeding status, anticipated poor compliance with study requirements, an age below 18 years, an inability to read or understand Danish, or a diagnosis of dementia.

### 3.2. Raman Measurement Protocol and Workflow

We used LaserSafe PC Professional Edition Ver 5.5 (Laser 2000), based on the standards IEC/EN 60825-1 and IEC/EN 60825-2, to calculate allowable laser power at the fiber probe exit. Bladder tissue is treated as skin for laser safety assessments. The maximum permissible exposure (MPE) was calculated for a multimode fiber (numerical aperture of 0.22) emitting at 785 nm in continuous wave mode for 0.5 s. At a 50 mW output power, the excess ratio of accessible emission to MPE for skin is 0.19. Due to a high beam divergence (14.7° with NA = 0.22), the nominal ocular hazard distance (NOHD) is only 17.3 cm, making it easy to maintain and eliminating the need for mandatory laser safety goggles. Moreover, the measurements were performed inside the bladder.

In this clinical study, up to four procedures per day were performed with the Raman invaScope system. This means that a minimum of five sterilized probes (one spare probe for risk mitigation) had to be available. Every time a new sterilized probe was used during cystoscopy, a calibration procedure was conducted before the in vivo Raman measurements. This calibration comprises the following two parts:

1. The wavenumber calibration of the spectrometer was performed using a polystyrene sample.
2. The intensity calibration of the detector was carried out using the NIST-certified SRM-2241, specifically designed for the 785 nm laser wavelength.

**Figure 7:**
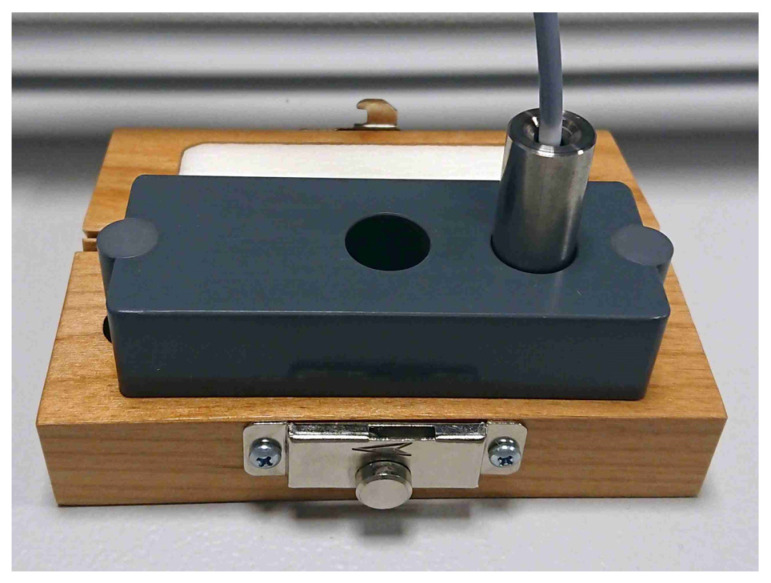
Calibration tool, based on a SRM 2241, with inserted fiber probe. The steel sheath is introduced to maintain sterility of the probe. The measurement on the right side allows us to perform a wavelength calibration, while the measurement on the left side enables the intensity calibration.

Unlike the Raman probe, the calibration samples used are non-sterile. To minimize contamination risk from touching non-sterile surfaces, a specialized calibration tool was developed for clinical application (Figure 7). This tool features a stainless-steel holder with a hole, which enables the insertion of the probe and ensures an appropriate spacing between the probe, the SRM-2241, and the polystyrene sample.

To maintain the sterility of the fiber probe, a sterile stainless-steel holder (included with each fiber probe) was also sterilized every time alongside the Raman probe. The intensity calibration serves to assess the performance of the fiber probe; a low-intensity reading can indicate transmission issues.

After system and software startup, patient, fiber probe, and surgeon identifiers have to be entered. The fiber probe was connected to the system and the calibration procedure was performed using the calibration tool described above. Meanwhile, the patient was prepared for a standard cystoscopy procedure under general anesthesia.

**Figure 8:**
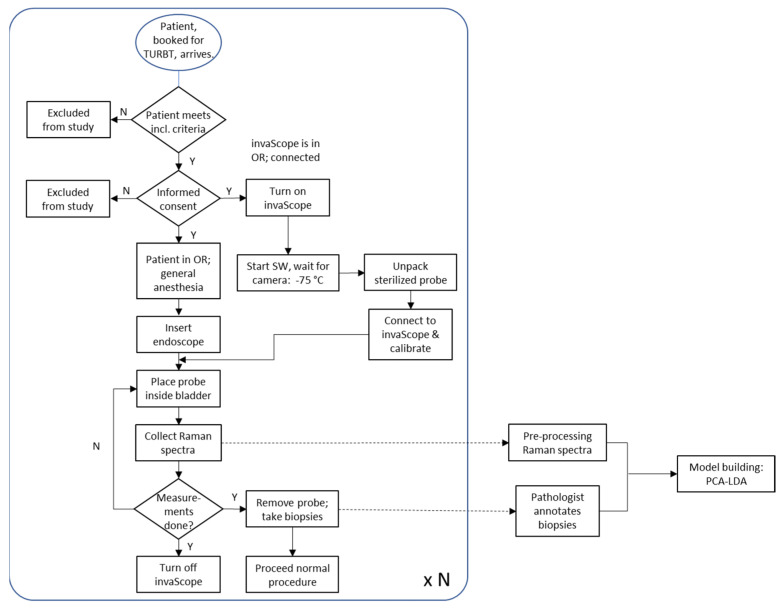
Summary of the entire procedure beginning with the booking of the patient for a TURBT, including the preparation of the patient for the procedure, the initialization of the invaScope, the measurements, and the pathological annotation and data processing. N—no; Y—yes; SW—software; OR—operation room.

**Figure 9:**
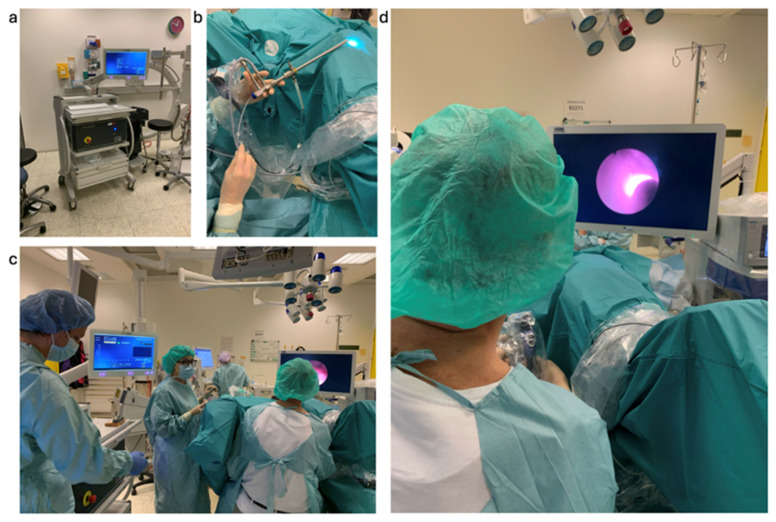
Raman invaScope system for clinical investigation of bladder cancer at the Herlev Hospital in Copenhagen. (**a**) invaScope in the surgical room before the measurements are taken on patients. (**b**) The endoscope (Blazejewski MEDI-TECH) which was used to access the bladder of the patient and to introduce the probe through the working channel. (**c**) Surgeons and clinical staff during the measurement procedure, where the invaScope is located on the left side of the image. (**d**) The excitation light from the fiber optic probe during the measurement in the bladder, as seen on the monitor of the endoscope.

After successful calibration, the urologist took the distal end of the probe and pushed it through the working channel of the endoscope into the bladder. Regions of interest (ROIs) were identified using white light endoscopy. The Raman fiber probe was moved to the ROI and brought into contact (light pressure) with the tissue. Raman spectral information was recorded using the predefined acquisition parameters (50 mW output power, 0.5 s acquisition time, no averaging to ensure laser safety, as mentioned above). Several spectra were recorded in one ROI. The fiber probe was moved to the next ROI and the Raman data acquisition was performed as before. This was repeated until spectra were collected from all identified ROIs, both from tumor tissue and normal mucosa. At the end of the Raman data collection, the fiber probe was removed from the endoscope and replaced with biopsy forceps. Biopsies for standard histopathological diagnosis were taken from both tumor tissue and normal mucosa. The aim was to take the biopsy at the site in the bladder where the Raman probe had previously been used for measurement. As the participating clinicians are very experienced, the reproducibility of the position in the bladder was sufficient for subsequent data evaluation. A standard TURBT was then performed. The workflow is summarized in the flowchart in Figure 8. The general setting for clinical measurements is presented in Figure 9.

With the outlined procedure, a total of 21 patients were examined. From these patients, an overall 44 biopsies were taken and 800 Raman spectra collected. Of the 800 measured Raman spectra, 438 spectra of 13 patients contained useful Raman spectral information (see Table A1). The other spectra had to be discarded due to the following reasons that could not be corrected for:

- Saturation of the detector due to high auto-fluorescence of the tissue.
- Interfering spectral features from the white or blue light sources of the endoscope used to illuminate the inner volume of the bladder. These light sources can be set to a low light intensity but cannot be easily switched off completely (during the procedure). Some of these light sources interfere with the Raman measurement, i.e., they are visible in the collected Raman spectra.
- Some patients were injected with hexaminolevulinate (Hexvix, [4,5,45]) prior to surgery. However, the use of Hexvix in the bladder can interfere with Raman spectroscopy. The strong fluorescence and Raman signals of Hexvix may overlap and interfere with Raman signals from the bladder tissue. Therefore, it is recommended that the dye is not used simultaneously with Raman experiments to avoid signal interference and maintain accurate spectroscopic data collection.

### 3.3. Biopsy Annotations

The biopsies were annotated by a trained pathologist according to the bladder cancer TNM-staging system ([2,3]) provided by the American Joint Committee on Cancer (AJCC) and the International Union Against Cancer (UICC). “T” refers to the size and extent of the primary tumor.

For this clinical study, six categories for biopsy annotations were defined (see Table 1).

### 3.4. Raman Spectral Data Analysis

**Figure 10:**
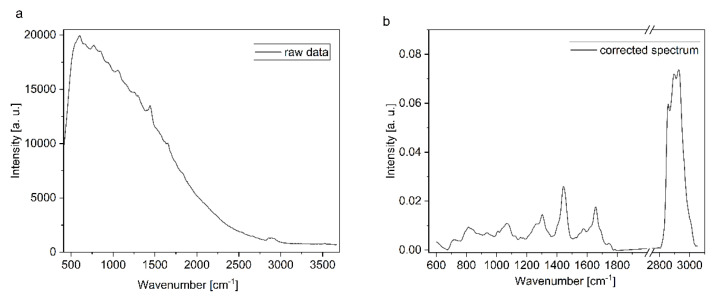
(**a**) Raw uncorrected in vivo Raman spectrum of bladder tissue, where a significant polynomial background is visible. (**b**) EMSC and background-corrected Raman spectrum after the intensity calibration and background correction.

Raman spectroscopy data processing and subsequent modeling were performed in R, a free statistical computing environment. To convert the spectral axis of the raw data from pixel values to the corresponding wavenumbers, the reference spectra of the polystyrene target with characteristic vibrational bands were used. In addition, an intensity correction was applied to account for the optical transfer function of the entire system based on the spectral profile of the NIST-certified SRM-2241 calibration standard. The wavenumber and intensity-calibrated spectra were corrected for unwanted contributions from the external light source using extended multiplicative scattering correction (EMSC, [46]). Briefly, an independently corrected target spectrum from the light source was used in an ordinary least squares fit procedure to estimate coefficients for the “spectral contaminant” to be subtracted. A polynomial baseline correction was then applied and finally, the spectra were normalized with respect to the region below the 2800–3100 cm^−1^ bands, which represent the C-H stretching vibrations of mainly proteins and lipids. Figure 10 shows a raw and an EMSC spectrum.

In some situations, there were remaining spectral contributions of the external light sources. Those spectra could not be used for Raman spectral analysis and had to be discarded (see above). This problem indicates that the external light source either has to be entirely switched off or the Raman fiber probe or system needs to be equipped with appropriate optical filters.

**Figure 11:**
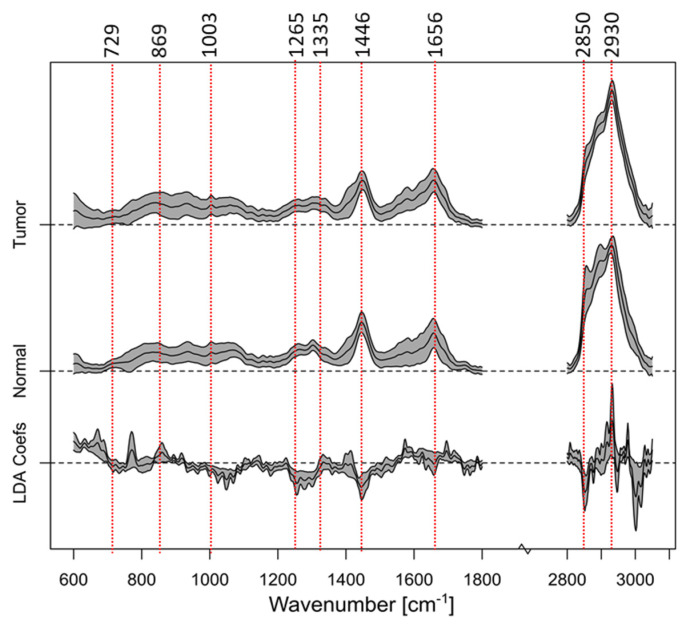
Results of the PCA–LDA classification process, where the average spectra for tumor and non-tumor tissue are presented as the mean with standard deviation. Furthermore, the LDA coefficients, which indicate the spectral contributions that are influencing the separation, are shown. The red dotted vertical lines indicate relevant band positions and are further described in Table 3.

A PCA–LDA classification [47] was performed to differentiate normal tissue from tumor tissue. The classification models were trained and validated using 10-fold cross-validation using internal and external test sets for estimating the optimal number of latent components. The results are displayed in Figure 11 and summarized in Table 2 in diagnostically relevant numbers. Table 2 reveals that tumor and non-tumor tissue can be differentiated with an accuracy of 83%, which is a very promising result and comparable to our ex vivo results. Band assignments and a thorough discussion of the differences between tumor and non-tumor tissue can be found in our ex vivo study on bladder cancer [18]. Based on this analysis, major bands are indicated in Figure 11 and summarized in Table 3.

**Figure 12:**
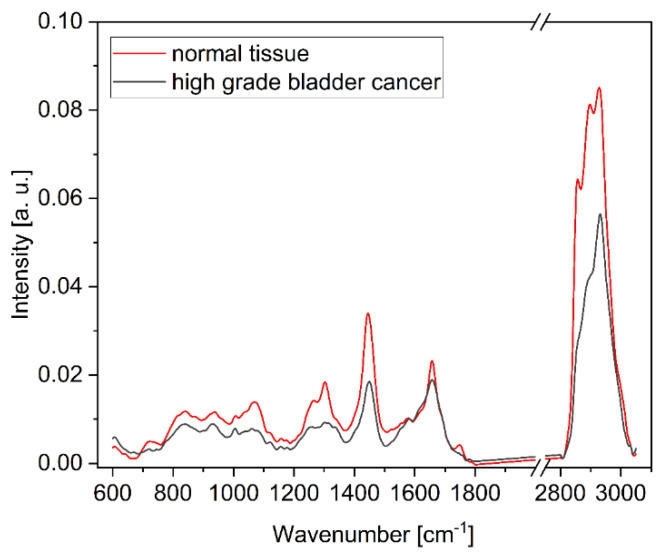
Corrected in vivo Raman spectra of healthy and high-grade tumor bladder tissue.

Even more important than differentiating between tumor and healthy surrounding tissue is the separation of low-grade and high-grade bladder cancer. Our ex vivo experiments have shown that Raman spectroscopy allows for a clear distinction between low- and high-grade tumors [18]. Figure 12 shows in vivo Raman spectra of normal- and high-grade tumor tissue. Table 4 shows the respective confusion matrix indicating an accuracy of 75% to separate low- from high-grade tumor tissue. Nevertheless, due to the low number of measurements for low-grade tissue and the resulting imbalance, these values are to be taken with caution. Further clinical studies are therefore needed, with a larger number of study participants and a more balanced ratio between the groups.

## 4. Discussion

In this work, we present the development and application of a Raman-based platform, invaScope, for application in bladder cancer diagnostics. This outline presents the journey from the bench to bedside while complying with the stringent European Medical Device Regulation. Our integration of the invaScope into existing resectoscope systems has demonstrated not only feasibility but also substantial potential to streamline and improve the accuracy of intraoperative cancer diagnostics in the bladder, as well as in other diseases. The clinical investigation outlined in this study successfully employed the invaScope to perform real-time in vivo Raman spectroscopy during TURBT procedures. Preliminary results highlight the probe’s capability to differentiate between tumorous and non-tumorous tissues effectively, potentially allowing urologists to achieve more precise tumor resections and tailor treatment strategies more effectively. This approach significantly reduces the diagnostic delay associated with conventional histopathology, thereby facilitating earlier treatment initiation and potentially improving patient outcomes.

A clinical study conducted at the Herlev Hospital in Copenhagen involved 21 patients with 44 individual measurement locations, where biopsies were also taken from. Based on the post-acquisition analysis, it was possible to achieve a sensitivity and specificity of 83% and 75% for the differentiation of tumor and normal tissue, respectively. For the differentiation between high-grade and low-grade tissue, a sensitivity of 77% and a specificity of 53% was achieved. The low and imbalanced number of measurements creates uncertainty; a larger study is needed to confirm these results. Nevertheless, those results correlate very well with our previous ex vivo findings. Due to challenges with the integrated illumination source in the resectoscope, not all measurements were useful, as the background contribution was too strong or the fluorescence signal from the tissue was too high. This was the initial clinical study conducted with the system. One objective was to identify potential issues not encountered in a laboratory setting or during ex vivo studies. One such issue was the interference from the emission of Hexvix and the light illumination of the endoscope. It became evident that a learning curve was required to optimize the workflow during operations and to obtain high-quality Raman spectra.

The influence of Hexvix requires further future investigation. Based on the present experience, Hexvix should be avoided during Raman-based investigations with a 785 nm excitation wavelength to minimize interference with clinical practice. To reduce fluorescence contributions, excitation wavelengths slightly above 785 nm, closer to 810 nm, could potentially be used to improve the outcome. This shift would lower excitation while maintaining detection in the high-wavenumber Raman region. Nevertheless, further investigation is required to determine optimal conditions for minimal interference from Hexvix. While our findings are promising, the path to routine clinical adoption remains challenging. Future studies should focus on validating the reproducibility of the invaScope in a larger cohort and across multiple institutions to confirm its robustness and reliability. To conduct multicenter studies, the presented invaScope must be replicated. This requires developing processes and strategies for characterization and calibration, as small, almost unavoidable differences between devices can significantly impact data evaluation. Specifically, model transfer between different devices may be compromised, making model-based separation prone to inaccuracies.

Additionally, further refinements in probe design and software algorithms are necessary to enhance the usability and integration into diverse clinical settings. A thinner probe, e.g., with an outer diameter of only 2 mm, would be compatible with flexible endoscopes that are used in the outpatient department. As presented in [48], outpatient flexible cystoscopy is less traumatic than the conventional inpatient procedure in the diagnosis of NMIBC. Another variant of our probe could be realized by adding a focusing element [23]. This would enable depth-selective Raman measurements, which could be advantageous for certain inflammation or cancer stages and for the identification of new biomarkers.

The different light sources present in the operating room influence the collected Raman signal to varying degrees, as discussed in [8]. Depending on the specific setting, suitable solutions must be found. Limiting the spectral range of the endoscope lamps applied can be a potential way of dealing with interfering light, while the influence of Hexvix on the Raman spectra requires further investigations.

For future investigation, it is also possible to harvest the full potential of Raman spectroscopy. For example, recent research has established a link between the urinary microbiome and microbial communities with the progression of bladder cancer. Specifically, the presence of certain bacteria, such as *Porphyromonas somerae*, has been identified as a potential indicator of the disease, suggesting a strong connection between microbial composition and cancer development [49]. These microbial interactions likely influence the tumor microenvironment through inflammatory mechanisms. A substantial body of work has readily demonstrated that Raman spectroscopy can differentiate specific bacteria. The combination of parallel microbiome and cancer characterization could not only enhance diagnostic accuracy but also open the door to personalized treatments that consider the dynamic relationship between microbial populations and tumor diagnostics.

## 5. Conclusions

In conclusion, the invaScope could represent a significant advancement for the real-time label-free diagnosis of bladder cancer. Our transition from the bench to bedside also presents the structural and institutional challenges stemming from the new medical device regulation, which should be addressed by the parliament to facilitate clearer clinical translation. Ultimately, our tool has the potential to offer urologists a powerful means to improve patient outcomes and reduce healthcare costs through enhanced diagnostic accuracy and treatment precision. Reducing the number of unnecessary biopsies (due to false positives) would significantly lessen the overall burden on patients. This study not only paves the way for future innovations in medical diagnostics but also sets a benchmark for compliance with new regulatory standards that govern medical device development and usage.

## Figures and Tables

**Table 1:** Categories of biopsy annotation as the pathological gold standard for Raman spectral data analysis.

Category	Description	Number of Biopsies
C1	pT2, muscle invasive cancer	1
C2	pTa (superficial tumor) with low-grade cells	7
C3	CIS (carcinoma in situ)	4
C4	Normal tissue	25
C5	pT1 (tumor in the soft mucosa) with high-grade cells	3
C6	pTa (superficial tumor) high-grade cells	4

**Table 2:** Confusion matrix for tumor and non-tumor classification. Columns list the predictions of the PCA–LDA model.

	Normal	Tumor
Normal	67	22
Tumor	12	104
Sensitivity	0.83	
Specificity	0.75	

**Table 3:** Raman band assignments for vibrational bands, which are indicated in Figure 11 and primarily responsible for the spectral separation of the tumor and non-tumor cases.

Wavenumber [cm^−1^]	Bond Assignment
729	C-C stretching, proline
869	C-C stretching, choline group
1003	Phenylalanine, C-C skeletal, phosphate group
1265	Amide III of collagen, v(CN), d(NH) amide III
1335	CH_3_CH_2_ wagging
1446	CH_2_ bending mode of proteins and lipids, CH_2_ deformation
1656	C-C lipids, amide I (proteins)
2850	υsCH_2_, lipids, fatty acids CH_2_ symmetric
2930	CH_2_ sym. stretching, chain-end CH_3_ sym. stretching

**Table 4:** Confusion matrix for low-grade and high-grade tumor classification. Columns list the predictions of the PCA–LDA model.

	High Grade	Low Grade
High grade	79	5
Low grade	23	7
Sensitivity	0.77	
Specificity	0.53	

## Data Availability

The datasets of this study are available from the corresponding author upon request.

## Appendix A

**Table A1:** Overview of the pathological data used for the Raman spectral data analysis.

	Hexvix Label	# Biopsies	# spc ^1^ Normal	# spc Low Grade (LG)	# spc High Grade (HG)
Patient 1	No	1	14	-	-
Patient 3	No	2	16	14	-
Patient 4	Yes	2	-	-	31
Patient 5	Yes	2	31	-	-
Patient 6	Yes	1	16	-	16
Patient 7	Yes	2	15	16	-
Patient 12	Yes	2	15	-	30
Patient 13	No	2	20	-	30
Patient 14	Yes	1	12	-	-
Patient 15	Yes	2	-	-	21
Patient 17	No	1	-	-	31
Patient 18	Yes	2	28	30	-
Patient 19	Yes	2	15	-	15
Total	-	22	182	60	174

^1^ # spc: number of spectra.

## References

[1] Zynger D.L. Staging-Bladder Carcinoma. https://www.pathologyoutlines.com/topic/bladderstaging.html.

[2] https://www.cancer.gov/about-cancer/diagnosis-staging/staging.

[3] Babjuk M., Bohle A., Burger M., Zigeuner R., Shariat S.F., van Rhijn B.W., Compérat E., Sylvester R.J., Kaasinen E., Böhle A. (2017). EAU Guidelines on Nonmuscle-Invasive Urothelial Carcinoma of the Bladder: Update 2016. Eur. Urol..

[4] Daneshmand S., Bazargani S.T., Bivalacqua T.J., Holzbeierlein J.M., Willard B., Taylor J.M., Liao J.C., Pohar K., Tierney J., Konety B. (2018). Blue light cystoscopy for the diagnosis of bladder cancer: Results from the US prospective multicenter registry. Urol. Oncol. Semin. Orig. Investig..

[5] Ray E.R., Chatterton K., Khan M.S., Chandra A., Thomas K., Dasgupta P., O’brien T.S. (2010). Hexylaminolaevulinate fluorescence cystoscopy in patients previously treated with intravesical bacille Calmette-Guerin. BJU Int..

[6] Mogensen K., Christensen K.B., Vrang M.-L., Hermann G.G. (2016). Hermann Hospitalization for transurethral bladder resection reduces quality of life in Danish patients with non-muscle-invasive bladder tumour. Scand. J. Urol..

[7] Auner G.W., Koya S.K., Huang C., Broadbent B., Trexler M., Auner Z., Elias A., Mehne K.C., Brusatori M.A. (2018). Applications of Raman Spectroscopy in Cancer Diagnosis. Cancer Metastasis Rev..

[8] Desroches J., Jermyn M., Mok K., Lemieux-Leduc C., Mercier J., St-Arnaud K., Urmey K., Guiot M.-C., Marple E., Petrecca K. (2015). Characterization of a Raman Spectroscopy Probe System for Intraoperative Brain Tissue Classification. Biomed. Opt. Express.

[9] Desroches J., Jermyn M., Pinto M., Picot F., Tremblay M.A., Obaid S., Marple E.T., Urmey K., Trudel D., Soulez G. (2018). A New Method Using Raman Spectroscopy for in Vivo Targeted Brain Cancer Tissue Biopsy. Sci. Rep..

[10] O’Brien C.M., Vargis E., Rudin A., Slaughter J.C., Thomas G., Newton J.M., Reese J., Bennett K.A., Mahadevan-Jansen A. (2018). In Vivo Raman Spectroscopy for Biochemical Monitoring of the Human Cervix throughout Pregnancy. Am. J. Obstet. Gynecol..

[11] Pence I.J., Beaulieu D.B., Horst S.N., Bi X., Herline A.J., Schwartz D.A., Mahadevan-Jansen A. (2017). Clinical Characterization of In Vivo Inflammatory Bowel Disease with Raman Spectroscopy. Biomed. Opt. Express.

[12] Cordero Bautista E., Latka I., Matthäus C., Schie I.W., Popp J. (2018). In-Vivo Raman Spectroscopy: From Basics to Applications. J. Biomed. Opt..

[13] Kerr L.T., Domijan K., Cullen I., Hennelly B.M. (2014). Applications of Raman Spectroscopy to the Urinary Bladder for Cancer Diagnostics. Photonics Lasers Med..

[14] Jin H., Lin T., Han P., Yao Y., Zheng D., Hao J., Hu Y., Zeng R. (2019). Efficacy of Raman Spectroscopy in the Diagnosis of Bladder Cancer. Medicine.

[15] Chen H., Li X., Broderick N., Liu Y., Zhou Y., Han J., Xu W. (2018). Identification and Characterization of Bladder Cancer by Low-Resolution Fiber-Optic Raman Spectroscopy. J. Biophotonics.

[16] Crow P., Molckovsky A., Stone N., Uff J., Wilson B.C., Wongkeesong L.M. (2005). Assessment of Fiberoptic Near-Infrared Raman Spectroscopy for Diagnosis of Bladder and Prostate Cancer. Urology.

[17] Bovenkamp D., Sentosa R., Rank E., Erkkilä M.T., Placzek F., Püls J., Drexler W., Leitgeb R.A., Garstka N., Shariat S.F. (2018). Combination of High-Resolution Optical Coherence Tomography and Raman Spectroscopy for Improved Staging and Grading in Bladder Cancer. Appl. Sci..

[18] Cordero Bautista E., Rüger J., Marti D., Mondol A.S., Hasselager T., Mogensen K., Hermann G.G., Popp J., Schie I.W. (2020). Bladder Tissue Characterization Using Probe-Based Raman Spectroscopy: Evaluation of Tissue Heterogeneity and Influence on the Model Prediction. J. Biophotonics.

[19] Placzek F., Cordero Bautista E., Kretschmer S., Wurster L.M., Knorr F., González-Cerdas G., Erkkilä M.T., Stein P., Ataman Ç., Hermann G.G. (2020). Morpho-Molecular Ex Vivo Detection and Grading of Non-Muscle-Invasive Bladder Cancer Using Forward Imaging Probe Based Multimodal Optical Coherence Tomography and Raman Spectroscopy. Analyst.

[20] Schie I.W., Placzek F., Knorr F., Cordero Bautista E., Wurster L.M., Hermann G.G., Mogensen K., Hasselager T., Drexler W., Popp J. (2021). Morpho-Molecular Signal Correlation between Optical Coherence Tomography and Raman Spectroscopy for Superior Image Interpretation and Clinical Diagnosis. Sci. Rep..

[21] Osterberg E.C., Laudano M.A., Li P.S. (2014). Clinical and Investigative Applications of Raman Spectroscopy in Urology and Andrology. Transl. Androl. Urol..

[22] Liu Y., Ye F., Yang C., Jiang H. (2024). Use of in Vivo Raman Spectroscopy and Cryoablation for Diagnosis and Treatment of Bladder Cancer. Spectrochim. Acta Part A Mol. Biomol. Spectrosc..

[23] Stomp-Agenant M., van Dijk T., Onur A.R., Grimbergen M., van Melick H., Jonges T., Bosch R., van Swol C. (2022). In Vivo Raman Spectroscopy for Bladder Cancer Detection Using a Superficial Raman Probe Compared to a Nonsuperficial Raman Probe. J. Biophotonics.

[24] Tanwar S., Paidi S.K., Prasad R., Pandey R., Barman I. (2021). Advancing Raman Spectroscopy from Research to Clinic: Translational Potential and Challenges. Spectrochim. Acta Part A Mol. Biomol. Spectrosc..

[25] Schleusener J., Gluszczynska P., Reble C., Gersonde I., Helfmann J., Cappius H.J., Fluhr J.W., Meinke M.C. (2015). Perturbation Factors in the Clinical Handling of a Fiber-Coupled Raman Probe for Cutaneous in Vivo Diagnostic Raman Spectroscopy. Appl. Spectrosc..

[26] Grimbergen M.C.M., van Swol C.F.P., van Moorselaar R.J.A., Uff J., Mahadevan-Jansen A., Stone N. (2009). Raman Spectroscopy of Bladder Tissue in the Presence of 5-Aminolevulinic Acid. J. Photochem. Photobiol. B Biol..

[27] Grimbergen M.C.M., van Swol C.F.P., Draga R.O.P., van Diest P., Verdaasdonk R.M., Stone N., Bosch J.H.L.R. (2009). Bladder Cancer Diagnosis during Cystoscopy Using Raman Spectroscopy. Photonic Ther. Diagn. V.

[28] Kong K., Kendall C., Stone N., Notingher I. (2015). Raman Spectroscopy for Medical Diagnostics—From in-Vitro Biofluid Assays to In-Vivo Cancer Detection. Adv. Drug Deliv. Rev..

[29] Barik A.K., Sanoop Pavithran M., Lukose J., Upadhya R., Pai M.V., Kartha V.B., Chidangil S. (2022). In Vivo Spectroscopy: Optical Fiber Probes for Clinical Applications. Expert Rev. Med. Devices.

[30] Cameron J.M., Rinaldi C., Rutherford S.H., Sala A., Theakstone A.G., Baker M.J. (2021). Clinical Spectroscopy: Lost in Translation?. Appl. Spectrosc..

[31] Stevens O., Petterson I.E.I., Day J.C.C., Stone N. (2016). Developing Fibre Optic Raman Probes for Applications in Clinical Spectroscopy. Chem. Soc. Rev..

[32] The European Union (2017). Regulation (EU) 2017/745 of the European Parliament and of the Council of 5 April 2017 on medical devices, amending Directive 2001/83/EC, Regulation (EC) No 178/2002 and Regulation (EC) No 1223/2009 and repealing Council Directives 90/385/EEC and 93/42/EEC. OJL.

[33] Shim M.G., Wilson B.C., Marple E.T., Wach M. (1999). Study of Fiber-Optic Probes for in Vivo Medical Raman Spectroscopy. Appl. Spectrosc..

[34] (2014). Standard Guide for Raman Shift Standards for Spectrometer Calibration.

[35] Choquette S.J., Etz E.S., Hurst W.S., Blackburn D.H., Leigh S.D. (2007). Relative Intensity Correction of Raman Spectrometers: NIST SRMs 2241 through 2243 for 785 Nm, 532 Nm, and 488 Nm/514.5 Nm Excitation. Appl. Spectrosc..

[36] (2020). Medical Electrical Equipment—Part 1–2: General Requirements for Basic Safety and Essential Performance—Collateral Standard: Electromagnetic Disturbances—Requirements and Tests.

[37] (2015). Industrial, Scientific and Medical Equipment—Radio-Frequency Disturbance Characteristics—Limits and Methods of Measurement.

[38] (1996). Medical Electrical Equipment—Part 1: General Requirements for Basic Safety and Essential Performance.

[39] (2013). Medical Electrical Equipment—Part 2–22: Particular Requirements for Basic Safety and Essential Performance of Surgical, Cosmetic, Therapeutic and Diagnostic Laser Equipment.

[40] (2014). Safety of Laser Products—Part 1: Equipment Classification and Requirements.

[41] (2017). Processing of Health Care Products—Information to Be Provided by the Medical Device Manufacturer for the Processing of Medical Devices.

[42] (2015). Medical Devices—Part 1: Application of Usability Engineering to Medical Devices.

[43] (2016). Medical Devices—Part 2: Guidance on the Application of Usability Engineering to Medical Devices.

[44] (2006). Medical Device Software—Software Life Cycle Processes.

[45] https://www.hexvix.com/safety-information.

[46] Afseth N.K., Kohler A. (2012). Extended Multiplicative Signal Correction in Vibrational Spectroscopy, a Tutorial. Chemom. Intell. Lab. Syst..

[47] Gautam R., Vanga S., Ariese F., Umapathy S. (2015). Review of Multidimensional Data Processing Approaches for Raman and Infrared Spectroscopy. EPJ Tech. Instrum..

[48] Mogensen K., Glenthøj A., Toft B.G., Scheike T., Hermann G.G. (2017). Outpatient photodynamic-guided diagnosis of carcinoma in situ with flexible cystoscopy: An alternative to conventional inpatient photodynamic-guided bladder biopsies in the operating theatre?. Scand. J. Urol..

[49] Nardelli C., Aveta A., Pandolfo S.D., Tripodi L., Russo F., Imbimbo C., Castaldo G., Pastore L. (2024). Microbiome Profiling in Bladder Cancer Patients Using the First-Morning Urine Sample. Eur. Urol. Open Sci..

